# Does personality affect health-related quality of life? A systematic review

**DOI:** 10.1371/journal.pone.0173806

**Published:** 2017-03-29

**Authors:** I-Chan Huang, Joy L. Lee, Pavinarmatha Ketheeswaran, Conor M. Jones, Dennis A. Revicki, Albert W. Wu

**Affiliations:** 1 Department of Epidemiology and Cancer Control, St. Jude Children’s Research Hospital, Memphis, Tennessee, United States of America; 2 Department of Medicine, School of Medicine, Indiana University, Indianapolis, Indiana, United States of America; 3 Herbert Wertheim College of Medicine, Florida International University, Miami, Florida, United States of America; 4 Outcomes Research, Evidera, Bethesda, Maryland, United States of America; 5 Department of Health Policy and Management, Johns Hopkins Bloomberg School of Public Health, Johns Hopkins University, Baltimore, Maryland, United States of America; National University of Singapore, SINGAPORE

## Abstract

**Background:**

Health-related quality of life (HRQOL) is increasingly measured as an outcome for clinical and health services research. However, relatively little is known about how non-health factors affect HRQOL. Personality is a potentially important factor, yet evidence regarding the effects of personality on HRQOL measures is unclear.

**Methods:**

This systematic review examined the relationships among aspects of personality and HRQOL. Eligible studies were identified from Medline and PsycINFO. The review included 76 English-language studies with HRQOL as a primary outcome and that assessed personality from the psychological perspective. Individuals with various health states, including ill (e.g., cancer, cardiovascular disorders), aging, and healthy, were included in this review study.

**Results:**

Some personality characteristics were consistently related to psychosocial aspects more often than physical aspects of HRQOL. Personality characteristics, especially neuroticism, mastery, optimism, and sense of coherence were most likely to be associated with psychosocial HRQOL. Personality explained varying proportions of variance in different domains of HRQOL. The range of variance explained in psychosocial HRQOL was 0 to 45% and the range of explained variance in physical HRQOL was 0 to 39%.

**Conclusions:**

Personality characteristics are related to HRQOL. Systematic collection and analysis of personality data alongside HRQOL measures may be helpful in medical research, clinical practice, and health policy evaluation.

## Introduction

Health-related quality of life (HRQOL) is increasingly used to evaluate treatment effectiveness be affected by psychological characteristics. Previous studies have begun to explore the relationships between personality characteristics and HRQOL [1–3]. Evidence indicates that adult personality tends to remain stable over long periods of time [4]. There is a general consensus that personality is a trait (a stable tendency to react a certain way) rather than a state (a reaction to an immediate situation). This distinction is of particular interest for research and evaluation, because personality influences an individual’s thoughts, feelings, and behaviors [5, 6].

Two personality measurement frameworks are commonly used to guide personality research: Eynsenck’s Three-Factor Model, which comprises neuroticism, extraversion, and psychoticism [7]; and the Five-Factor Model, which comprises neuroticism, extraversion, agreeableness, conscientiousness, and openness to experience [8]. These models capture the personality traits that the authors believe to be essential and orthogonal. A limitation of both models is that they emphasize superordinate traits rather than subordinate traits (or facets) that might be of interest to researchers [9]. Another approach to delineating personality is to focus on individual traits that may be incorporated into one of the superordinate factors of the models but also have their own specific focus. Individual traits typically considered in medical research include optimism (expecting good things will be plentiful in the future and bad things will be scarce), aggression (attempting to harm another person), hopefulness (tendency to construct and respond to the perceived future positively), negative affectivity (disposition to subjective distress), and sense of coherence (confidence that one’s internal and external environment are predictable and that there is a high probability things will work out).

Two different approaches have been used by investigators to study the relationships between personality and HRQOL. One approach examines the direct effect of specific personality traits on HRQOL [1, 4, 9]. Other approach emphasizes the functional aspects of personality, for example examining how personality influences health through perceptions, cognition, values, goals adjustment, motivations, biological factors, and behaviors [10, 11].

A greater understanding of the relationships between personality and health could enhance research on the effectiveness of health care interventions and treatments, by increasing the amount of variance in patient outcomes that can be explained. It could also help physicians identify barriers to treatment adherence and subsequently improve their patients’ health outcomes. For example, optimistic people adhere better to treatment regimens, use adaptive coping to reduce physiological consequences of stress, and report fewer stressful events, fewer somatic symptoms, and better functional status than pessimistic patients [12–14]. However, to our knowledge, there has not been a comprehensive review of the influence of personality on HRQOL.

We conducted a systematic review of literature on the relationships among personality characteristics and dimensions of HRQOL. We aimed to identify the magnitude of personality characteristics associated with different domains of HRQOL, investigate the potential mechanisms through which personality affects HRQOL, and examine the amount of variance in HRQOL that is explained by specific personality characteristics.

## Methods

### Literature search strategy

**Fig 1:**
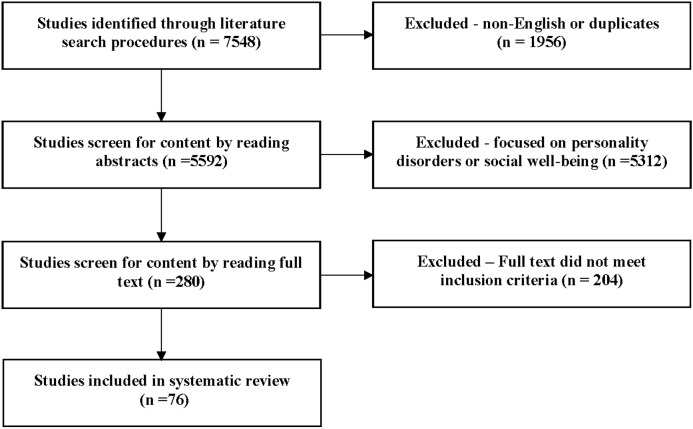
Flowchart of studies selected to be included in the systematic review.

We used PubMed and PsycINFO to identify relevant studies using the medical subject heading (MeSH) keywords “quality of life” and “personality” (Fig 1). To identify additional studies, we applied 26 of the most widely used HRQOL measures [15] as key words. References cited in the identified articles were examined to obtain additional studies for further consideration. We restricted the search to English-language articles published between January 1, 1985, and December 31, 2009.

### Inclusion and exclusion criteria for study selection

Different terms related to personality traits have appeared in the literature. The inclusion of specific personality traits to this study is based on the two most commonly used frameworks (Eynsenck’s Three-Factor Model [7] and the Five-Factor Model [8]) and the consensus among our team members after reviewing the literature on the association of personality trait and HRQOL. The definitions for more specific personality characteristics and related measures are listed in S1 Appendix. We included studies that used standard psychological measures of personality that possess acceptable measurement properties (i.e., reliability and content and construct validity) [16]. We excluded studies that treated personality measures as diagnostic criteria for psychopathology [15], and those that focused on social or subjective well-being [17, 18] since this construct is distinct from HRQOL. Additionally, we excluded studies that explored the relationships between personality and HRQOL solely for the purpose of validating HRQOL measures. For validation studies, developers might further revise HRQOL measures; therefore, the psychometric properties of HRQOL measures are not always optimal. Finally, we excluded studies that reported personality characteristics and HRQOL, but did not examine the relationships between the two variables.

Two investigators (ICH, AWW) independently reviewed the abstract of each study to confirm the eligibility. If an abstract was selected as eligible, the same authors independently reviewed the respective articles to confirm that they met the inclusion criteria. Discrepancies were adjudicated by consensus, or failing this, by other investigators (PK, MAA).

### Data extraction and analysis

We designed a data extraction form to extract information from each study on the specific aims, population, settings, design, methods, domains of personality, domains of HRQOL, and major findings. The study design was categorized as cross-sectional (CS) versus longitudinal cohort (CO). We categorized HRQOL domains into overall/global QOL, psychological functioning/well-being, role functioning, social functioning, vitality, physical functioning, bodily pain, general health perceptions, somatic symptoms, and other functioning. We further classified these domains as either physical aspects or psychosocial aspects of HRQOL [19].

We analyzed and reported the findings based on the relationships between personality characteristics and HRQOL. Specifically, we examined the magnitude of bivariate association between individual personality characteristics and HRQOL variables using correlation coefficients (r) and effect sizes (Cohen’s d), and examined the variance in HRQOL explained by personality characteristics (R^2^). We also examined separately the statistical significance of the relationships conducted by t-test, analysis of variance (ANOVA), linear regression analysis, logistic regression analysis, path analysis, or structural equation modeling, and reported the percentage of analyses that demonstrated a significant relationship between personality and HRQOL domains. We defined the percentage of significant results as the number of analyses with statistically significant findings (p-value <0.05) divided by the number of analyses identified from the studies under our review.

**Fig 2:**
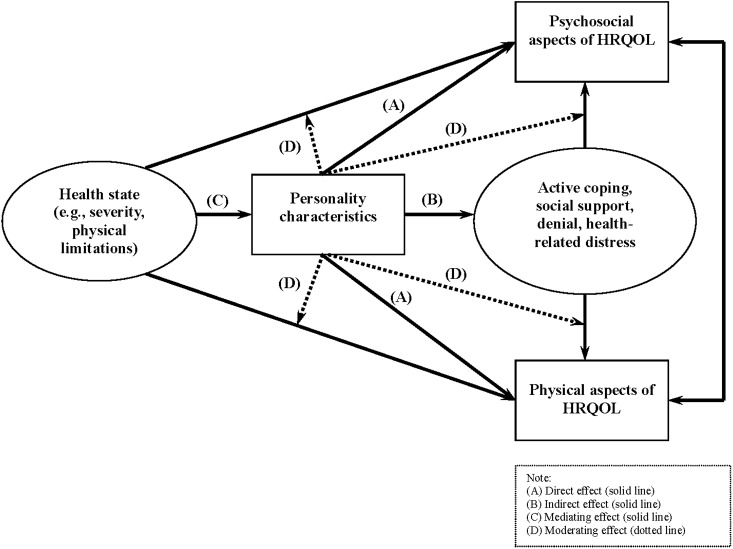
Pathways of personality to psychosocial aspects and physical aspects of HRQOL.

We hypothesized that personality characteristics would be more strongly associated with psychosocial aspects than physical aspects. A schematic of the potential mechanisms through which personality influences HRQOL is shown in Fig 2, in which the specific mechanisms were classified as direct (Route A), indirect (Route B), mediating (Route C), and moderating effects (Route D). We generated this personality-HRQOL conceptual framework up front to guide our analyses.

Indirect effects were defined as the influence of personality characteristics on HRQOL through the effects of other variables, such as social support or coping style. Path analysis or structural equation modeling was generally used to identify the presences of indirect effects [20].

Mediating effects occur when an independent variable, such as disease severity, affects HRQOL by acting through the influence of the effect of a personality characteristic. Ideally, this effect can be tested using datasets containing changes in independent variables, personality traits, and HRQOL. In this review study, we categorized an effect as mediating if 1) there was a significant association between personality and the independent variable, 2) there was a significant association between personality and HRQOL, and 3) the association between the independent variable and HRQOL diminished subsequently after adjusting for the effect of personality variables. In contrast, moderating effects were defined as the relationships between HRQOL and personality characteristics that differ depending upon a third variable, such as a stressful event. An interaction term of personality with the third variable is usually tested to identify the moderating effects. If no moderating effects exist, then we can regard personality variables as independent predictors of HRQOL [21].

We investigated whether personality characteristics were more likely to affect HRQOL reported by patients themselves or that reported by proxies (e.g., family members, physicians or observers). We hypothesized that personality would be more strongly associated with self-ratings of HRQOL than with proxy-ratings for the patients, and personality characteristics would associate with the discrepancy in HRQOL rated by patients themselves and their proxies.

Finally, we investigated the importance of personality characteristics on HRQOL relative to other factors (e.g. sociodemographic or biomedical) by examining the standardized coefficients of regression analysis or structural equation models, and the amount of variance explained in the reviewed studies.

## Results

### Characteristics of the selected studies

We initially identified 7,548 studies through literature search procedures, of which 1,956 were excluded because they were non-English or duplicates. Of the 5,592 remaining studies, we excluded an additional 5,312 studies after reviewing the abstracts either because they did not investigate the association between personality characteristics and HRQOL, they focused on personality disorders or social well-being rather than HRQOL, or they were designed to validate HRQOL measures. Of the 280 remaining studies, we excluded 204 after reviewing the full-text articles based on the review inclusion criteria (Fig 2). Table 1 shows the characteristics of 76 studies under our review in a chronological order from newest to oldest; these studies included a total of 336 individual statistical analyses investigating the relationships between personality characteristics and HRQOL.

**Table 1:** Characteristics of reviewed studies of personality characteristics and HRQOL.

Author [Reference #]	Country	Disease-specific population	Sample Size	SD	Personality trait	Personality measure	HRQL domain	HRQL measure
Badura-Brzoza et al [22]	Poland	Total hip replacement	102	CO	State and trait anxiety, sense of coherence, neuroticism, and extraversion	STAI, SOC, and EPI	Generic (BP, PF, RP, RE, MH, SF, VT, GH, PCS, and MCS)	SF-36
Bartoces et al [23]	USA	Cervical cancer	145	CS	Self esteem	RSE	Generic (PCS, and MCS)	SF-36
Dubayova et al [24]	Slovakia	Parkinson's disease	153	CS	Type D personality and negative affectivity	DS-14	Condition-specific (overall QOL, mobility, activities of daily living, emotional well-being, stigma, social support, cognition, communication, and bodily discomfort)	PDQ-39
Dubayova et al [25]	Slovakia	Parkinson's disease	153	CS	Neuroticism and extraversion	EPQ-R	Condition-specific (mobility, activities of daily living, emotional well-being, stigma, social support, cognition, communication, and bodily discomfort)	PDQ-39
Kall et al [26]	Sweden	Whiplash associated disorders	40	CO	Self-efficacy	SES	Generic (BP, PF, RP, RE, MH, SF, VT, GH, PCS, and MCS)	SF-12
Kobayashi et al [27]	Japan	Cervical cancer	60	CS	Self-esteem	RSE	Condition-specific (physical, social/family, emotional, and functional)	FACT-G
Kvarme et al [28]	Norway	School children	279	CS	Self-efficacy	GSE	Generic (physical well-being, emotional well-being, self-esteem, and friends)	KINDL
Siassi et al [29]	Germany	Post major colorectal surgery	79	CO	Neuroticism, extraversion, agreeableness, and sense of coherence	NEO-FFI and SOC	Generic (BP, PF, RP, RE, MH, SF, VT, GH, PCS, and MCS) Condition-specific (overall QOL)	SF-36 and GLQI-39
Tsaousides et al [30]	USA	Adults under 65 with traumatic brain injury	425	CS	Self-efficacy	IND/BBQ	Overall QoL	LLATBI
Visser-Meily et al [31]	Netherlands	Post aneurysmal subarachnoid hemorrhage	141	CS	Neuroticism	EPQ	Condition-specific (emotional functioning)	SSQOL
Warrian et al [32]	USA	Glaucoma	147	CS	Neuroticism, extraversion, openness, agreeableness, and conscientiousness	NEO-PI-R	Condition-specific (overall QOL, general vision, mental health, social functioning, role limitation, and ocular pain)	VFQ
Aarstad et al [33]	Norway	Head and neck squamous cell carcinoma	55	CO	Neuroticism, extraversion, and lie	EPI	Condition-specific (global QOL, functioning scale, cancer symptom scale, and head and neck cancer symptom scale)	EORTC QLQ-H&N35 and EORTC-QLQ-C30
Boye et al [34]	Norway	Ulcerative colitis and Crohn's disease	110	CS	Aggression, alexithymia, neuroticism, and lie	BPA, EPQ, and TAS	Condition-specific (overall QOL, emotional functioning, social functioning, systemic symptoms, and bowel functioning)	IBDQ
Boye et al [35]	Norway	Ulcerative colitis and Crohn's disease	109	CS	Aggression, alexithymia, neuroticism, and lie	BPA, EPQ, and TAS	Generic (BP, PF, RP, RE, MH, SF, VT, and GH)	SF-36
De Bolle et al [36]	Belgium	Children with cancer	54	CS	Neuroticism, extraversion, imagination, benevolence, and conscientiousness	HiPIC	Generic (overall QOL, PF, EF, SF, and SC)	PedsQL 4.0
Jerant et al [37]	USA	Chronic conditions	245	CS	Neuroticism, extraversion, openness, agreeableness, and conscientiousness	NEO-FFI	Generic (overall QOL, mobility, self-care, usual activities, pain/discomfort, and anxiety/depression)	EQ-5D
Aquarius et al [38]	Netherlands	Peripheral arterial disease	150	CS	Type D personality	DS-14	Generic (overall QOL, physical health, and level of independence)	WHOQOL-100
Aquarius et al [39]	Netherlands	Peripheral arterial disease	203	CO	Type D personality	DS-14	Generic (BP, PF, RP, RE, MH, SF, VT, and GH)	RAND-36
Chapman et al [40]	USA	Elderly	442	CS	Neuroticism, extraversion, openness, agreeableness, and conscientiousness	NEO-FFI and SOC	Generic (SF, RE, RP, and PF)	SF-36
Cohen et al [41]	Israel	Adolescents with heart disease	173	CS	Self esteem	RSE	Condition-specific (limitations)	TAAQOL-CHD
Karlsson et al [42]	Sweden	Coronary artery disease	224	CO	Type D personality, sense of coherence, and negative affectivity	DS-24 and SOC	Generic (overall QOL)	Cantril Ladder of Life
Middleton et al [43]	Australia	Spinal cord injuries	106	CS	Self-efficacy	Moorong and SES	Generic (BP, PF, RP, RE, MH, SF, VT, GH, PCS, and MCS)	SF-36
Moreno-Jimenez et al [44]	Spain	Crohn's disease and ulcerative colitis	120	CS	Neuroticism	EPI-N and RSE	Condition-specific (overall QOL, emotional function, social function, systemic symptoms, and bowel function)	IBDQ
Pedersen et al [45]	Netherlands	Post implantation of cardiverter-defibrillator	154	CO	Type D personality	DS-14	Generic (BP, PF, RP, RE, MH, SF, VT, and GH)	SF-36
van der Steeg et al [46]	Netherlands	Breast disease	202	CO	Neuroticism, extraversion, openness, and state-trait anxiety	NEO-PI-R and STAI	Generic (overall QOL, physical health, psychological health, and social relationships)	WHOQOL-100
Arnold et al [47]	Netherlands	Chronic obstructive pulmonary disease	39	CO	Self-efficacy	Sullivan and SES	Generic (PF, MH, SF, and overall)	SF-36 and Cantril Ladder or Life
Friedman et al [48]	USA	Breast cancer	81	CS	Optimism	LOT	Condition-specific (physical well-being, emotional well-being, and functional well-being)	FACIT-G
Hantash et al [49]	Palestine	Teeth implant	50	CO	Neuroticism, extraversion, openness, agreeableness, and conscientiousness	NEO-FFI	Condition-specific (overall QOL, appearance, pain, oral comfort, general performance, and eating ability)	DIDL
Ong et al [50]	Canada	Atrial fibrillation	93	CS	Optimism	LOT	Generic (BP, PF, RP, RE, MH, SF, VT, GH, PCS, and MCS)	SF-36
Pedersen et al [51]	Netherlands	Heart transplantation	188	CS	Type D personality	DS-14	Generic (BP, PF, RP, RE, MH, SF, VT, GH, PCS, and MCS)	SF-36
Aquarius et al [52]	Netherlands	Peripheral arterial disease	300	CS	Type D personality	DS-14	Generic (overall QOL,physical health, and level of independence)	WHOQOL-100
van den Berg et al [53]	Netherlands	Paroxysmal atrial fibrillation	73	CS	Neuroticism	EPQ-R	Generic (BP, PF, RP, RE, MH, SF, VT, and GH)	SF-36
Vollrath et al [54]	Norway	Pediatric patients with unintentional injuries	107	CO	Neuroticism, extraversion, openness, agreeableness, and conscientiousness	HiPIC	Generic (overall QOL, physical complaints, motor functioning, autonomy, cognitive functioning, social functioning, positive emotions, and negative emotions)	TACQOL
Zhang et al [55]	China	Liver transplant candidates	55	CS	Neuroticism, extraversion, psychoticism, and lie	EPQ	Generic (physical, material, social, and psychological well-being)	GQOLI-74
De Clercq et al [56]	Belgium	Pediatric cancer survivors	67	CS	Neuroticism, extraversion, openness, agreeableness, and conscientiousness	HiPIC	Generic (overall QOL, physical functioning, emotional functioning, social functioning, and school functioning)	PedsQL 4.0
Sears et al [57]	USA	Implantable cardioverter defibrillator	88	CO	Optimism	LOT	Generic (MH, SF, and GH)	SF-36
Wittkowski et al [58]	UK	Atopic dermatitis	125	CS	Self esteem	RSES	Condition-specific (work, leisure, relationships, daily activities and treatment)	DLQI
Aarstad et al [59]	Norway	Head and neck squamous cell carcinoma	96	CS	Neuroticism, extraversion, and lie	EPI	Condition-specific (global QOL, functioning scale, cancer symptom scale, and head and neck cancer symptom scale)	EORTC QLQ-H&N35 and EORTC-QLQ-C30
Duberstein et al [60]	USA	Aging primary care patients	265	CS	Neuroticism, extraversion, openness, agreeableness, and conscientiousness	NEO-FFI	Generic (PF, GH)	SF-36
Han et al [61]	Korea	Chronic conditions	1748	CS	Self-efficacy and self-esteem	RSE and SES	Generic (physical, psychological, and socio-economic)	Ro's
Penedo et al [62]	USA	HIV/AIDS	116	CS	Neuroticism, extraversion, openness, agreeableness, and conscientiousness	NEO-PI-R	Condition-specific (overall function, life satisfaction, medical worries, financial worries, medication worries, HIV master, disclosure worries, provider trust, and sexual functioning)	HAT-QOL
Cormier et al [63]	France	Prostate cancer screening	334	CS	Trait-anxiety	STAI	Generic (BP, PF, RP, RE, MH, SF, VT, GH, PCS, and MCS)	SF-36
Goodwin et al [64]	USA	General population	3606	CS	Neuroticism, extraversion, conscientiousness, openness, and agreeableness	MIDI	Generic (overall subjective health perception)	Newly-developed health perception scale
Rose et al [65]	Germany	Diabetes	625	CS	Optimism	SWOP	Generic (overall QOL)	WHOQOL-BREF
Wasylkiw et al [66]	Canada	Psychology students	350	CS	Neuroticism, extraversion, conscientiousness, openness, and agreeableness	NEO-PI	Generic (PF, RP, RE, MH, SF, VT, and GH)	SF-36
Cederfjall et al [67]	Sweden	HIV	189	CS	Sense of coherence	SOC	Generic (overall QOL) and condition-specific (HIV symptoms)	HI and self-developed HIV symptom Scale
Kressin et al [68]	USA	General population	691	CS	Negative affectivity	EPI-Q	Condition-specific (oral HRQOL, psychosocial functioning, physical functioning, and pain)	OHQOL, OHIP, and GOHAI
Tanum et al [69]	Norway	Functional gastrointestinal disorder	111	CS	Neuroticism, extraversion, conscientiousness, openness, and agreeableness, and hostility	EPQ and NEO-PI	Generic (pain)	McGill Pain Questionnaire
Achat et al [70]	USA	General population	659	CO	Optimism	LOT	Generic (BP, PF, RP, RE, MH, SF, VT, GH, PCS, and MCS)	SF-36
Allison et al [71]	France	Head and neck cancer	101	CO	Optimism	LOT	Condition-specific (overall QOL, functioning scale, and symptom scale)	EORTC-QLQ-C30
Burgess et al [72]	UK	HIV seropositive	279	CS	Neuroticism, extraversion, and psychoticism	EPQ-R	Condition-specific (role functioning, physical functioning, health distress, and mental health)	MOS-HIV
Canizares et al [73]	Spain	Epilepsy	33	CO	Neuroticism	EPQ-A	Condition-specific (cognitive functioning)	QOLIE-31
Denollet et al [74]	Holland	Coronary heart disease	319	CO	Type D personality	DS-16	Generic (perceived health symptoms and disability)	HCS and GMS
Fritz [75]	USA	Heart attack or check pain	65	CO	Agency, communion, and unmitigated communion	PAQ and HUCS	Generic (MCS and PCS)	SF-36
Kempen et al [76]	Holland	Congestive heart failure and acute myocardial infarction	213	CS	Neuroticism	EPQ-R	Generic (functional disability	GARS
Kressin et al [77]	USA	General population	1629	CS	Negative affectivity	EPI-Q	Generic (BP, PF, RP, RE, MH, SF, VT, GH, PCS, and MCS)	SF-36
Nesbitt et al [78]	USA	Elderly	137	CS	Sense of coherence	SOC	Generic (Overall QOL)	Ferrans and Power Quality of Life Index-General Version
Spiro III et al [18]	USA	General population	1257	CS	Extraversion	EPI-Q	Generic (PF, RP, BP, GH, VT, SF, RE, MH) and condition-specific (cognitive functioning)	SF-36 and MHI
Scott et al [79]	USA	Orthognathic surgery	117	CO	Neuroticism	EPI-Q	Generic (psychosocial scale) and condition-specific (general oral health, effect on work and social activities, oral esthetics, pain/sensitivity, and oral functioning)	SIP and OHSQ
Yamaoka et al [80]	Japan	Stomach cancer	828	CS	Neuroticism, extraversion, and psychoticism	EPQ	Generic (positive and negative HRQOL)	HRQOL-20 (Japanese version)
Zhu et al [81]	China	Epilepsy	201	CS	Neuroticism, extraversion, and psychoticism	EPQ	Generic (general physical health)	General well-being Schedule
Carr et al [82]	Australia	Earthquake victims	845	CO	Neuroticism, and extraversion	EPI	Generic (psychological distress)	GHQ-12
Hollifield et al [83]	USA	Panic attack	123	CS	Neuroticism	NEO-FFI	Generic (PF, MH, and GH)	SF-36
Kempen et al [84]	Holland	Elderly	5279	CS	Neuroticism	EPQ-R	Generic (PF, RF, SF, HP, BP, and MH)	SF-20
Wettergren et al [85]	Sweden	Malignant blood disorders	20	CO	Sense of coherence	SOC	Condition-specific (overall QOL, functioning scale, and symptom scale)	EORTC-QLQ-C30
Chen et al [86]	UK	Suspicion of breast cancer	121	CO	Neuroticism and extraversion	EPI	Generic (psychological distress)	GHQ-12
Joukamaa et al [87]	Finland	Elderly	190	CO	Alexithymia	TAS-26	Generic (psychological distress)	GHQ-32
Kempen et al [88]	Holland	Elderly	624	CO	Neuroticism, extraversion	EPQ	Generic (motor, hearing, and vision functioning)	OECD indicator, HHDI-D, and a self-developed scale for vision.
Kempen et al [89]	Holland	Elderly	753	CO	Neuroticism, extraversion, and mastery	EPQ	Generic (functioning activity)	GARS-ADL subscale
Hidding et al [90]	Holland	Ankylosing spondylitis	67	CO	Neuroticism	DPI	Generic (global health)	SIP, HAQ-S, Functional Index for Ankylosing spondylitis, and self-developed hospital visual analogue scale
Carver et al [91]	USA	Breast cancer	70	CO	Optimism	LOT	Generic (subjective well-being, sex quality, and pain)	Profile of Mood States, Life Satisfaction Scale, Quality of Sex Life, and self-developed pain scale
Hidding et al [92]	Holland	Ankylosing spondylitis	144	CS	Neuroticism	DPI	Generic (overall QOL) and Condition-specific (activities of daily living, physical functioning, and psychosocial functioning)	SIP, HAQ-S, and SAF
Jorm et al [93]	Australia	Elderly	711	CS	Neuroticism and extraversion	EPQ-R	Generic (Overall QOL, physical functioning, vision, hearing, pain, activity of daily living, and symptom)	Self-developed scales
Hooker et al [94]	USA	Caregivers of Alzheimer disease or dementia	51	CS	Neuroticism and optimism	NEO-FFI and LOT	Generic (mental and physical HRQOL)	Bradbun Affect Balance, HPQ- Current Health Subscale, self-developed health index, and MAI
Wilson et al [95]	UK	Globus sensation	46	CS	Neuroticism, extraversion, and lie	EPI	Generic (psychological distress)	GHQ-60
Scheier et al [96]	USA	Psychology students	141	CO	Optimism	LOT	Generic (physical symptoms)	Physical Symptom Checklist- 39

SD: Study Design.

**CO:** cohort study.

**CS:** cross-sectional study.

Personality Measures.

**BPA**: Buss-Perry Aggression Questionnaire.

**DPI:** Dutch Personality Inventory.

**DS-14**: Type D Scale 14.

**DS-16**: Type D Scale 16.

**DS-24**: Type D Scale 24.

**EPI**: Eysenck Personality Inventory.

**EPI-Q**: Short form of Eysenck Personality Inventory Emotional Stability Scale.

**EPQ**: Eysenck Personality Questionnaire.

**EPQ-A**: Eysenck Personality Questionnaire – Spanish Adult version.

**EPQ-R**: Eysenck Personality Questionnaire Revised.

**GSE**: Generalized Self-Efficacy Scale.

**HiPIC**: Hierarchical Personality Inventory for Children.

**HUCS**: Helgeson’s Unmitigated Communion Scale.

**IND/BBQ**: Independence (IND) subscale of the Bigelow Quality of Life Questionnaire (BBQ).

**LOT**: The Life Orientation Test.

**MIDI**: Midlife Development Inventory Personality Scales.

**NEO-PI**: The Neuroticism, Extraversion and Openness Personality Inventory.

**NEO-PI-R**: The Neuroticism, Extraversion and Openness Personality Inventory Revised.

**NEO-FFI**: The Neuroticism, Extraversion and Openness Five-Factor Inventory- short version.

**PAQ**: Personal Attributes Questionnaire.

**RSE:** Rosenberg Self Esteem scale.

**SES:** Self-Efficacy Scale.

**SOC**: Antonovsky’s Sense of Coherence Questionnaire.

**STAI**: State and Trait Anxiety Inventory.

**SWOP**: The Assessment of Beliefs in Self-Efficacy and Optimism.

**TAS:** Toronto Alexithymia Scale.

HRQOL Measures

**DIDL**: Dental Impact on Daily Living.

**EORTC-QLQ-C30**: European Research and Treatment of Cancer Quality of Life Core Questionnaire.

**EORTC-QLQ-H&N35**: European Research and Treatment of Cancer Quality of Life Questionnaire module for patients with head and neck cancer.

**EQ-5D**: EuroQol-5 Dimension questionnaire.

**FACIT-G**: The Functional Assessment of Chronic Illness Therapy-General.

**GARS**: Groningen Activity Restriction Scale.

**GHQ-12/GHQ-32/GHQ-60**: General Health Questionnaire 12-/ 32-/60-item versions.

**GOHAI**: Geriatric Oral Health Assessment Instrument.

**GLQI-39**: Gastrointestinal Quality of Life Index.

**GMS**: Global Mood Scale.

**GQOLI-74**: General Quality of Life Inventory.

**HAQ-S:** Health Assessment Questionnaire for the Spondyloarthropathies

**HAT-QOL**: The HIV/AIDS-Targeted Quality of Life Instrument.

**HCS**: The Health Complaints Scale.

**HHDI-D:** Hearing Handicap and Disability Inventory – Disability Subscale.

**HI:** Health Index.

**HPQ:** Health Perceptions Questionnaire.

**HRQOL-20**: Health Related Quality of Life questionnaire.

**IBDQ**: Inflammatory Bowel Disease Questionnaire

**KINDL**: A generic instrument for assessing HRQOL in children and adolescents.

**LLATBI**: The Living Life After Traumatic Brain Injury.

**MAI**: Multilevel Assessment Instrument.

**MHI**: Mental Health Index.

**MOS-HIV**: Medical Outcomes Study-HIV Health Survey.

**OHIP**: Oral Health Impact Profile.

**OHQOL**: Oral Health Related Quality of Life.

**OHSQ**: Oral Health Status Questionnaire.

**PedsQL 4.0**: Pediatric Quality of Life Inventory version 4.0.

**PDQ-39**: Parkinson’s Disease Questionnaire.

**QOLIE-31**: Quality of Life in Epilepsy Inventory.

**RAND-36**: RAND 36-Item Health Survey.

**SAF:** Self-Assessed Function questionnaire.

**SF-20/SF-36**: The Short Form 20-Item/36-Item Health Survey.

**SIP:** Sickness Impact Scale.

**SSQOL**: Stroke-Specific Quality of Life.

**TACQOL**: TNO-AZL Children’s Quality of Life questionnaire.

**VFQ**: National Eye Institute's Visual Function Questionnaire.

**WHOQOL-100**: World Health Organization Quality of Life Assessment.

HRQOL Domains of the SF-36/ RAND-36/SF-20.

**PF**: Physical functioning.

**RP**: Role limitation due to physical problems.

**BP**: Bodily pain.

**GH**: General health perceptions.

**VT**: Vitality

**SF**: Social functioning.

**RE**: Role limitation due to emotional problems.

**MH**: Mental health.

Among personality measures, 45 studies (59%) assessed neuroticism (including negative affectivity) using either the Eysenck Personality Inventory/Questionnaire (EPI/EPQ), the NEO-Personality Inventory (NEO-PI), the NEO Five-Factor Inventory (NEO-FFI), the Dutch Personality Inventory (DPI), HiPIC, MIDI, DS-14, or DS-24; 29 (38%) assessed extraversion, and 9 assessed optimism (12%) (Table 1). Other personality characteristics that were included by >5 studies were openness to experience, agreeableness, conscientiousness, self-esteem, self-efficacy, and Type D personality. S1 Appendix summarizes the definitions of personality dimensions, the corresponding traits, and tools to assess personality traits.

Regarding HRQOL measures, 23 studies (30%) used generic and condition-specific measures derived from the Medical Outcomes Studies (MOS), including the SF-36/SF-20 (21 studies), the RAND-36 (one study), and the MOS-HIV (one study). Of other generic measures, 4 studies (5%) used the General Health Questionnaire, 3 (4%) used the Sickness Impact Profile (SIP), and 4 (5%) used the World Health Organization Quality of Life Assessment Instrument (WHOQOL). Of other condition-specific measures, 4 studies (5%) used the European Research and Treatment of Cancer Quality of Life Questionnaire (EORTC-QLQ) (Table 1).

### Relationships between personality characteristics and HRQOL

All of the studies found the relationships between specific personality characteristics and HRQOL to be in the same direction. For example, greater extraversion, agreeableness, openness, conscientiousness, optimism, self-esteem, self-efficacy, and sense of coherence were all related to better HRQOL, while greater neuroticism, negative affectivity, and type D personality were related to poorer HRQOL (Table 2).

**Table 2:** Relationships between personality dimension, single personality trait, and HRQOL.

Personality dimensions	Overall or global QOL	General health perception	Psychological functioning	Physical functioning	Role functioning	Social functioning	Other specific functioning	Vitality	Bodily pain	Somatic symptom
Agreeableness	● [56]^‡^ ○ [32]^‡^, [36]^‡^, [37]^‡^	● [64]^‡^ ○ [64]^‡^ ^,^ [60]^‡^	○ [32]^‡^, [36]^‡^	○ [36]^‡^, [40]^‡^ [60]^‡^	○ [32]^‡^, [40]^‡^	● [36]^‡^, ○ [32]^‡^, [40]^‡^	○ [32]^‡^, [36]^‡^		● [49]^‡^ ○ [32]^‡^	
Conscientiousness	● [32]^‡^, ○ [36]^‡^, [37]^‡^, [56]^‡^	● [64]^‡^ ○ [60]^‡^	○ [32]^‡^, [36]^‡^	○ [36]^‡^, [40]^‡^ [60]^‡^	● [40]^‡^ ○ [32]^‡^	○ [32]^‡^, [36]^‡^, [40]^‡^	● [32]^‡^ ○ [36]^‡^		○ [32]^‡^	
Extraversion	● [32]^‡^, [81]^‡^ ○ [25]^‡^, [36]^‡^, [37]^‡^, [56]^‡^	● [64]^‡^ ○ [60]^‡^	○ [22]^‡^, [25]^‡^, [36]^‡^, [64]^‡^, [72]^‡^, [18]^‡^, [82]^‡^, [86]^‡^	● [18]^‡^, ○ [22]^‡^, [25]^‡^, [36]^‡^, [40]^‡^, [72]^‡^ [60]^‡^	○ [32]^‡^, [40]^‡^	● [32]^‡^ ○ [25]^‡^, [36]^‡^, [40]^‡^	● [25]^‡^, [88]^‡^, ○ [25]^‡^, [32]^‡^, [36]^‡^, [88]^‡^		○ [25]^‡^, [32]^‡^	
Neuroticism	● [25]^‡^, [33]^†^^‡^, [34]^‡^, [37]^‡^, [56]^‡^, [79]^‡^, [81]^‡^, [92]^‡^ ○ [32]^‡^, [36]^‡^, [90]^‡^	● [53]^‡^, [64]^‡^, [83]^‡^, [84]^‡^ [60]^‡^ ○ [35]^‡^, [36]^‡^	● [22]^‡^, [25]^‡^, [31]^‡^, [32]^‡^, [34]^‡^, [35]^‡^, [44]^‡^, [53]^‡^, [63]^‡^, [18]^‡^, [82]^‡^, [83]^‡^, [84]^‡^, [86]^‡^, [25]^‡^, [33]^‡^ ○ [22]^‡^, [36]^‡^	● [22]^‡^, [18]^‡^, [94]^‡^ ○ [22]^‡^, [25]^‡^, [35]^‡^, [36]^‡^, [40]^‡^, [53]^‡^, [72]^‡^, [76]^‡^, [83]^‡^, [84]^‡^ [60]^‡^	● [40]^‡^ ○ [32]^‡^, [35]^‡^, [53]^‡^, [84]^‡^	● [25]^‡^, [40]^‡^, [44]^‡^, [53]^‡^, [84]^‡^ ○ [32]^‡^, [34]^‡^, [35]^‡^, [36]^‡^	● [25]^‡^, [33] ^†^^‡^, [44]^‡^, [59]^†^, [73]^‡^, [79]^‡^, [88]^‡^ ○ [25]^‡^, [32]^‡^, [34]^‡^, [36]^‡^, [88]^‡^	● [35]^‡^ ○ [53]^‡^	● [25]^‡^, [33]^†^^‡^, [84]^‡^ ○ [32]^‡^, [53]^‡^, [69]^‡^	● [33]^†^^‡^, [44]^‡^, [59]^†^ ○ [34]^‡^
Openness to experience	● [56]^‡^ ○ [32]^‡^, [36]^‡^, [37]^‡^	● [64]^‡^ ○ [60]^‡^	○ [32]^‡^, [36]^‡^,	● [60]^‡^ ○ [36]^‡^, [40]^‡^	○ [32]^‡^, [40]^‡^	● [40]^‡^ ○ [32]^‡^, [36]^‡^	● [36]^‡^ ○ [32]^‡^		○ [32]^‡^	
Psychoticism	● [81]^‡^									
%, significant results	46%	50%	50%	17%	15%	36%	29%	50%	33%	75%
Single Personality Trait										
Agency			● [75]^‡^	○ [75]^‡^						
Aggression	○ [34]^‡^		○ [34]^‡^, [35]^‡^		● [35]^‡^	○ [34] ^‡^	○ [34]^‡^		● [69]^‡^	○ [34]^‡^,
Alexithymia	○ [34] ^‡^		○ [34]^‡^, [35]^‡^, [87]^‡^	● [35]^‡^	● [35]^‡^	● [35]^‡^ ○ [34]^‡^	○ [34]^‡^	○ [35]^‡^		● [87]^‡^
Communion			○ [75]^‡^	○ [75]^‡^						
Dispositional optimism	● [65]^‡^, [71]^‡^	● [70]^‡^ ○ [57]^†^	● [48]^‡^, [50]^‡^, [57]^†^, [70]^‡^, [71]^‡^ ○ [94]^‡^	○ [50]^‡^, [70]^‡^, [71]^‡^, [94]^‡^	● [94]^‡^ ○ [70]^‡^	● [48]^‡^, [57]^†^, ○ [70]^‡^, [71]^‡^	● [48]^‡^ ○ [71]^‡^	● [70]^‡^	● [70]^‡^, [71]^‡^	● [71]^‡^
Hopefulness			● [82]^‡^							
Lie	● [34]^‡^		○ [22]^‡^, [34]^‡^	● [35]^‡^ ○ [22]	○ [35]^‡^	●[34]^‡^	○ [34]^‡^		○ [35]^‡^	○ [34]^‡^
Negative affectivity	● [68]^‡^	● [77]^‡^	● [68]^‡^, [77]^‡^	● [68]^‡^ ○ [77]^‡^	● [77]^‡^	● [68]^‡^, [77]^‡^	● [68]^‡^	● [77]^‡^	● [68]^‡^, [77]^‡^	
Self-efficacy	● [26]^‡^, [28]^‡^	● [43]^†^	● [43]^†^, [44]^‡^, [28]^‡^	● [43]^†^, [28]^‡^	● [43]^†^	● [43]^†^, [28]^‡^ ○ [44]^‡^	● [44]^‡^	● [43]^†^	● [43]^†^	
Self-esteem	● [27]^†^, [41]^‡^, ○ [58]^‡^		●[23]^‡^, [27]^†^	○ [23]^‡^, [27]^†^		● [27]^†^				
Sense of Coherence	● [67]^‡^, [78]^‡^	● [42]^†^	● [22]^‡^ ○ [22]^‡^	● [22]^‡^ ○ [22]^‡^						● [67]^‡^,
Trait-anxiety	● [46]^‡^	● [63]^‡^	● [22]^‡^, [46]^‡^, [63]^‡^ ○ [36]^‡^	● [22]^‡^, [46]^‡^ ○ [22]^‡^, [63]^‡^	○ [63]^‡^	● [46]^‡^, [63]^‡^		● [63]^‡^	○ [63]^‡^	
Type-D	● [24]^‡^, [38]^^^, [45]^†^, [52]^^^, [74]^^^	● [39]^†^, [42]^†^ ○ [51]^^^	● [24]^‡^, [39]^†^, [51]^^^, [74]^‡^	● [38]^^^, [51]^^^, [52]^^^ ○ [24]^‡^, [39]^†^	● [39]^†^, [51]^^^	● [24]^‡^, [39]^†^, [51]^^^	● [24]^‡^, [38]^^^, [52]^^^	● [39]^†^, [51]^^^	● [39]^†^, ○ [24]^‡^, [51]^^^	
Unmitigated communion			○ [75]^‡^	○ [75]^‡^						
%, significant results	84%	78%	65%	39%	70%	74%	60%	86%	64%	60%

● Statistically significant.

○ Not statistically significant.

† t-test/ ANOVA.

‡ Multivariate regression analysis.

^ Multivariate (odds ratio) analysis.

The level of significance of the relationships between personality and HRQOL varied depending upon the type of personality characteristics and domains of HRQOL measured (Table 2). A total of 75% of the statistical analyses showed significant associations between personality characteristics and vitality, 54% with social functioning, and 58% with psychological functioning. Only 28% of analyses showed significant associations with physical functioning (Table 2). A total of 60% of analyses showed significant associations with general health perception, and 57% with overall quality of life (Table 2).

The magnitude of correlation coefficients (in absolute value) between personality and specific domains of HRQOL ranged from 0.04 to 0.74; the magnitude of effect sizes (in absolute value) between personality and the specific domain of HRQOL ranged from 0 to 4.2 (Table 3). Studies consistently showed that personality characteristics were more likely to be associated with psychosocial aspects (e.g. psychological functioning, vitality, and social functioning) than physical aspects of HRQOL (e.g. physical functioning, role limitation due to physical problems, or bodily pain) (Table 3). For the MOS questionnaires, the correlation coefficients (in absolute value) of personality characteristics with mental component scores (MCS) were larger than with physical component scores (PCS); 0.29–0.64 versus 0.28–0.34.

**Table 3:** The strength of relationships between personality characteristics and HRQOL.

Personality characteristics	Correlation coefficient^¶^		Effect size^§^	
Agency [75]	• PCS	• 0.28		
Aggression [34, 35]	• Psychological functioning • Role limitation- emotional • Social functioning • Overall QOL	• 0.26-0.43 (0.33) • 0.38 • 0.24 • 0.24-0.31		
Agreeableness [29, 40, 49, 54, 60, 66]	• General health perceptions • Role limitation- physical • Role limitation- emotional • Social functioning • PCS • MCS • Overall QOL • Oral functioning	• 0.10 • 0.13 • 0.22 • 0.18 • 0.32-0.34 • 0.37 • 0.18-0.22 • 0.35		
Alexithymia [34, 35]	• Physical functioning • Role limitation- physical • Psychological functioning • Role limitation- emotional • Vitality • Social functioning	• 0.33 • 0.29 • 0.26-0.49 • 0.25 • 0.25 • 0.30		
Conscientiousness [32, 40, 54, 60, 62, 66]	• Physical functioning • Role limitation- physical • Psychological functioning • Role limitation- emotional • General health perceptions • Social functioning • Overall QOL • Cognitive functioning • HIV functioning • Sexual functioning • Visual functioning	• 0.18 • 0.19-0.28 • 0.27 • 0.22-0.29 • 0.16-0.24 • 0.21-0.26 • 0.24-0.25 • 0.28 • 0.20 • 0.22 • 0.19	• Role limitation- physical	• 0.12
Extraversion [18, 29, 40, 55, 59, 60, 62, 66, 80, 81, 82, 88, 93]	• Physical functioning • Role limitation- physical • Psychological functioning • Role limitation- emotional • General health perceptions • Bodily pain • Vitality • Social functioning • MCS • Overall QOL • Cognitive functioning • HIV functioning • Sexual functioning • Visual functioning • Hearing functioning	• 0.12-0.39 (0.26) • 0.16-0.25 • 0.18-0.26 (0.20) • 0.10-0.26 • 0.17-0.21 • 0.08-0.15 (0.15) • 0.25 • 0.08-0.29 (0.28) • 0.32-0.50 (0.46) • 0.10-0.47 (0.27) • 0.19 • 0.30 • 0.31 • 0.10 • 0.12		
Mastery [89]	• Performance-based measure	• 0.20		
Negative affectivity [77]	• Physical functioning • Psychological functioning	• 0.17 • 0.67	• Role limitation- physical • Psychological functioning • Role limitation- emotional • General health perceptions • Bodily pain • Vitality • Social functioning • MCS	• 0.14-0.20 • 0.25-0.50 (0.44) • 0.16-0.30 (0.27) • 0.12-0.14 • 0.06-0.21 • 0.15-0.18 • 0.16-0.17 • 0.22-0.43 (0.37)
Neuroticism 25, 29, 31-35, 37, 40, 44, 49, 54, 55, 59, 60, 62, 66, 73, 80-84, 86, 88, 92-95]	• Physical functioning • Role limitation- physical • Psychological functioning • Role limitation- emotional • General health perceptions • Bodily pain • Vitality • Social functioning • MCS • Overall QOL • Role functioning • Cognitive functioning • Oral functioning • HIV functioning • Hearing functioning • Visual functioning • Sexual functioning	• 0.12-0.39 (0.28) • 0.10-0.33 (0.24) • 0.21-0.67 (0.53) • 0.12-0.47 (0.28) • 0.17-0.54 (0.32) • 0.23-0.46 (0.31) • 0.20-0.48 (0.37) • 0.14-0.44 (0.29) • 0.44-0.58 (0.48) • 0.04-0.71 (0.33) • 0.13-0.40 (0.21) • 0.23-0.55 (0.45) • 0.40-0.54 • 0.39 • 0.10-0.24 • 0.23-0.27 • 0.27	• Physical functioning • Psychological functioning • Role limitation- emotional • General health perceptions • Social functioning • Overall QOL	• 0 • 1.4 • 0.34 • 0.6 • 0.22 • 0.16
Psychoticism [55, 81]	• Psychological functioning • Overall QOL	• 0.40 • 0.40		
Self-efficacy [26, 28, 30, 43, 61]	• Overall QOL • Physical functioning • Social functioning • Psychological functioning • Bodily pain • Role functioning • Vitality • General Health • Somatic Symptom • PCS/MCS	• 0.39-0.40 • 0.54 • 0.64 • 0.41 • 0.38 • 0.41-0.43 • 0.60 • 0.64 • 0.40 • 0.64-0.68	• Physical functioning • Psychological functioning • Social functioning	• 0.21 • 0.26 • 0.30
Self-esteem [23, 41, 58, 61]	• Overall QOL	• 0.35-0.47 (0.38)	• MCS	• 0.11
Sense of coherence [29, 78, 85]	• Physical functioning • Role functioning • Social functioning • MCS • Overall QOL	• 0.40-0.68 • 0.46 • 0.62-0.64 • 0.56-0.64 (0.61) • 0.42-0.74 (0.58)		
Openness [40, 54, 60]	• Physical functioning • Role limitation- emotional • Social functioning • Cognitive functioning	• 0.11-0.13 • 0.17 • 0.20 • 0.33	• Social functioning	• 0.16
Optimism [48, 50, 70, 91, 94, 96]	• Physical functioning • Psychological functioning • Social functioning • PCS • MCS	• 0.22-0.54 (0.31) • 0.37- 0.55 (0.37) • 0.40 • 0.30 • 0.38-0.50 (0.45)	• Physical functioning • Role limitation- physical • Psychological functioning • Role limitation- emotional • General health perceptions • Bodily pain • Vitality • Social functioning • PCS • MCS	• 0.01-1.60 • 0.01-1.70 • 0.03-2.40 • 0.03-2.20 • 1.80-4.21 • 1.72-2.00 • 2.40-2.61 • 0.01-1.90 • 1.60 • 2.50
Lie [55]	• Psychological functioning	• 0.32		
Unmitigated communion [75]	• MCS	• 0.29		

^¶^ Correlation coefficient reported for statistically significant results; absolute value reported; median reported in parentheses (where available).

^§^ Effect size = unit change in HRQOL scores for 1 standard deviation unit change in personality variable; absolute value reported; median reported in parentheses (where available)

Examination of specific personality characteristics suggested that neuroticism, negative affectivity, and sense of coherence were more likely than other characteristics to correlate with psychosocial aspects of HRQOL. Neuroticism and sense of coherence were moderately correlated with MCS, with absolute correlation coefficients of 0.44–0.58 and 0.56–0.64, respectively. There was a strong correlation between sense of coherence and social functioning and between negative affectivity and psychological functioning, with absolute correlation coefficients 0.62–0.64 and 0.67, respectively. In contrast, the correlation coefficients of agreeableness, extraversion, and optimism with MCS were 0.37, 0.32–0.50, and 0.38–0.50, respectively.

### Self-ratings vs. proxy-ratings of HRQOL

Personality characteristics were more likely to be associated with self-ratings of HRQOL than proxy-ratings of HRQOL [88, 89, 93]. For example, Kempen et al. showed that the significant association between the patient’s self-rating of dressing/getting around the house and mastery was maintained after adjusting for the proxy-rating of motor and hearing functioning [89]. Personality characteristics were also associated with a discrepancy between self- and proxy-ratings of HRQOL [88, 89]. For example, patients with lower mastery and extraversion were also likely to report lower scores in self-rating of dressing, getting around the house, and standing, compared to proxy-ratings [89].

### Impact on the change of HRQOL

This review study includes 27 studies that collected data across multiple time points. However, only three studies investigated if personality at baseline can predict the change in HRQOL over time, and the remaining 24 studies measured HRQOL at a follow-up time point alone. These three studies found that personality can influence the change of HRQOL. Aquarius et al. found that among patients with peripheral arterial disease, type-D personality was related to more impaired HRQOL over time than those with non-type-D personality [39]. Allison et al. found that cancer patients who were optimistic were more likely to improve their global and role HRQOL than their pessimistic counterparts [71]. Interestingly, Hidding et al. reported that after group physical therapy, ankylosing spondylitis patients with low self-esteem tended to improve more in global HRQOL than those with high self-esteem [90].

### Significant associations of other factors with HRQOL

Personality characteristics were stronger determinants of HRQOL than sociodemographic factors such as age [67], social integration [78] and income [78] as well as clinical factors such as comorbidity [78], CD4+ counts and HIV disease stage [67, 72] and seizure outcome after epilepsy surgery [73].

### Specific effects of personality characteristics on HRQOL

Personality characteristics had indirect, mediating, and moderating effects on different aspects of HRQOL (Table 4). Eight studies [33, 50, 65, 72, 75, 78, 79, 94] provided evidence that personality characteristics were indirectly associated with HRQOL, that is, personality characteristics affected HRQOL through another variable, such as coping style [33, 72], social support [72], stress [94], and doctor-patient relationships [65]. One study found that the effects of physical disability on HRQOL were mediated by sense of coherence [78]. Three studies [71, 82, 84] examining moderating effects of personality on HRQOL demonstrated that personality could modify the effects (or strength of the effect) of other variables on HRQOL. Neuroticism modified the effect of an earthquake stressor on psychological distress [82] and the effects of chronic conditions on physical and social functioning [84]. Optimism modified the effect of duration of disease on overall QOL and role functioning [71].

**Table 4:** Specific effect of personality characteristics on HRQOL.

Personality characteristics	Indirect effect	Mediating effect	Moderating effect	Reference
Neuroticism	On QOL scores through coping efforts			[33]
Optimism	On mental QOL and psychological distress through symptom preoccupation			[50]
Optimism	On overall QOL through doctor-patient relationships			[65]
Optimism			Optimism modifies time effect (duration of disease) on role functioning and overall QOL	[71]
Neuroticism	On psychological HRQOL through coping style and social support			[72]
Unmitigated communion, communion, and agency	Unmitigated communion: on psychological HRQOL through instrumental constrains and failure to adhere an exercise regimen			[75]
Sense of coherence	On HRQOL though illness appraisal	Mediates the effect of physical health limitation on HRQOL		[78]
Neuroticism	On oral HRQOL through psychological distress and psychological functioning affect			[79]
Neuroticism			Neuroticism modifies the effect of earthquake stressor on psychological distress	[82]
Neuroticism			Neuroticism modifies the effect of chronic condition on health perception, physical functioning, and social functioning;	[84]
Neuroticism and extraversion			Neuroticism and extraversion modifies the effect of aging on general health perceptions	[60]
Neuroticism and optimism	Neuroticism: on mental HRQOL through perceived stress. Optimism: on mental and psychical HRQOL through perceived stress			[94]

### Additional variance in HRQOL explained by personality characteristics

Personality characteristics explained varying proportions of variance in HRQOL, depending on the specific personality types and domains of HRQOL measured (Table 5). As expected, personality characteristics explained greater variance in psychosocial aspects of HRQOL than physical aspects. Variance explained in psychosocial HRQOL was often >10%, with a range between 0 and 45%. In contrast, the variance in physical HRQOL explained was a range between 0 and 39%. For overall QOL, the variance explained by personality ranged between 1 and 40%. Comparing the variance explained by single personality characteristics, Hooker et al. found that neuroticism explained 39% of the variance in psychological HRQOL, but only 17–29% in physical HRQOL [94].

**Table 5:** Additional variance in HRQOL explained by personality characteristics.

Personality characteristics	Variance explained in HRQOL % by personality characteristics	Reference
Negative affectivity	9-13% (overall QOL)	[24]
Negative affectivity	0.1-4% (pain), 1-12% (physical functioning), 1-12% (overall QOL), 10% (social functioning), 4-8% (psychological functioning), 3-18% (psychosocial functioning)	[68]
Negative affectivity	0% (physical function), 0-2% (vitality), 0-2% (social functioning), 0-1% (pain), 0-1% (general health perception), 1-3% (role physical functioning), 2-5% (role emotional functioning), 3-14% (mental functioning)	[77]
Neuroticism	10% (overall QOL)	[33]
Neuroticism	7% (overall QOL)	[54]
Neuroticism	17-25% (overall QOL), 30% (emotional functioning)	[59]
Neuroticism	Neuroticism: 25% (overall QOL)	[92]
Neuroticism and extraversion	Neuroticism: 1% (overall QOL); extraversion: 11% (overall QOL)	[36]
Neuroticism and optimism	Neuroticism: 39% (mental HRQOL), 17-29% (physical HRQOL); optimism: 34% (mental HRQOL), 10-19% (physical HRQOL)	[94]
Neuroticism and lie	Neuroticism: 36% (overall QOL), 23% (emotional functioning); lie: 15% (overall QOL)	[34]
Neuroticism, extraversion, conscientiousness, openness, and agreeableness	Combined personality traits: 12% (child self-rated social functioning), 36% (child self-rated school functioning), 36% (parent rated emotional functioning), 26% (parent-rated social functioning), 14% (parent rated school functioning), and 18% (parent-rated overall QOL)	[35]
Neuroticism, extraversion, conscientiousness, openness, and agreeableness	38% (child self-rated overall QOL), 16% (parent-rated overall QOL)	[56]
Neuroticism, extraversion, conscientiousness, openness, and agreeableness	Combined personality traits: 0% (physical functioning); 6% (role physical functioning); 12% (social functioning); 12% (role emotional functioning); 14% (general health perception); 28% (vitality); 45% (mental health)	[66]
Neuroticism, extraversion, conscientiousness, openness, and agreeableness	3% (physical functioning), 4% (general health perceptions)	[60]
Sense of coherence	28% (overall QOL)	[78]
Mastery	6.5% (Performance-based measure)	[89]
Optimism	21% (MCS), 41% (psychological distress)	[50]
Self-efficacy	40% (overall QOL)	[26]
Self-efficacy	16% (overall QOL)	[30]
Self-esteem	5% (overall QOL)	[61]
Trait anxiety	24-39% (physical functioning), 18% (psychological functioning)	[46]

## Discussion

We conducted a systematic review of 76 studies that examined the relationship of personality characteristics to HRQOL. Personality appears to have consistent relationships with HRQOL that are moderate in magnitude and often outweigh the effects of demographic, social, and even clinical factors. However, personality is more often related to psychosocial aspects of HRQOL than to physical aspects. The magnitude of correlation coefficients between personality characteristics and specific domains of HRQOL ranged from 0.04 to 0.74 (median = 0.30) and the effect sizes ranged from 0 to 4.2 (median = 0.18). Variance explained in psychosocial HRQOL was between 0 and 45% (median = 11%), whereas the variance in physical HRQOL was between 0 and 39% (median = 2%). In particular, neuroticism, negative affectivity, and sense of coherence generally show moderate correlations with psychosocial HRQOL. Neuroticism was most likely to be related to psychological functioning; for example, 39% of the variance in psychological HRQOL versus 17–29% in physical HRQOL was explained by neuroticism [94]. Few studies have examined the impact of personality traits on the longitudinal change of HRQOL. It is evident that individuals with type-D [39] and pessimism [71] personality, respectively, possess higher risk of consistently impaired HRQOL over time than those with non-type-D and optimism personality.

As expected, personality characteristics were more strongly related to patients’ self-rating of their own HRQOL than were proxy-ratings made on their behalf. Interestingly, personality characteristics also predicted the discrepancy between self- and proxy-ratings of functioning. For example, patients with higher neuroticism and lower extraversion and mastery were likely to self-report more impaired hearing, motor, and ADL functioning than their proxies reported for them [88, 89, 97]. This suggests that personality may bias the proxy ratings of patients’ health status.

Although explorations of the mechanisms between personality and health are increasing, existing theories and models focus on the outcomes of health state/illness [16], subjective well-being (SWB) [98], and death [99–101] rather than HRQOL. Fig 2 illustrates the possible links between personality characteristics and HRQOL based on the rubric of trait-related theories explaining the direct effects of personality on HRQOL and the rubric of cognitive behavior-related theories explaining the indirect effects.

Trait theory emphasizes the effect of common genetic biological factors [102, 103]. There is evidence that genetic factors explain 30%-60% of the variance in personality traits [104] and 40% of the variance in HRQOL. Trait theory explains individual differences from two perspectives (emotional reactivity and cognitive processing of information), which helps explain how personality is related to HRQOL [105]. The emotional reactivity hypothesis [106] suggests that the differences in an individual’s well-being may be due to differences in emotional reactivity, which are governed by personality traits. For example, extraverts may react more strongly to pleasant emotional stimuli than introverts, and may be more likely to experience pleasant affect when exposed to a positive event. The cognitive processing of information hypothesis [107] suggests that individuals are more likely to perceive trait-congruent information than incongruent information. For example, extraverts were quicker to relate events to their motives when they were in a positive mood, whereas introverts were quicker when in a negative mood [107]. Therefore, personality characteristics influence both what information is processed and how it is interpreted. The proposed direct links between personality characteristics and HRQOL (route A in Fig 2) reflect the results of the majority of the studies under our review (also see specific studies listed in Tables 2 and 3). This direct links incorporate both schools of thought to suggest that personality influences emotional reactivity and the cognitive schema through which individuals perceive, interpret, and encode their internal somatic experience and HRQOL.

Trait-related theories, unfortunately, cannot explain the mechanisms by which personality influence HRQOL [108]. Cognitive behavior theory (or the Transactional model) emphasizes that personality affects the illness appraisal and influences individuals’ coping process [16]. Although this theory was initially applied to SWB rather than HRQOL, illness appraisal behavior is a significant process influencing HRQOL, and the conclusions can be extended to HRQOL. This theory focuses on two aspects of individual differences (congruence and goal adjustment). The congruence hypothesis proposes that individuals may experience high HRQOL when they engage in behaviors (e.g., coping) that are concordant with their personality traits [105]. The goal adjustment hypothesis argues that individuals have a global tendency to experience HRQOL in a way determined by their personality [105]. Individuals with a personality characterized of being able to disengage from unattainable goals and reengage elsewhere are able to support active coping and avoid failure experiences, consequently maintaining a higher HRQOL [9]. Based on cognitive behavior-related theories, the linkages (route B in Fig 2) suggest that the effects of personality characteristics on HRQOL might be mediated by social support, coping skill, health behaviors (e.g. smoking, drug abuse), or psycho-physiological mechanisms [70, 79, 94, 109, 110].

It is also possible that personality *mediates* the effect of other variables (e.g. severity and physical limitations) on HRQOL (route C in Fig 2). Although personality traits have a considerable hereditary component and are less modifiable than HRQOL [111], some evidence suggests individuals who experience traumatic events (e.g., crime and hurricane [112]) or deteriorating health states (e.g., stroke, respiratory disease [113, 114] or cancer [115]) may trigger the change of personality to some extent. Recent studies even suggest that individuals who experience traumatic events may encounter positive personality change (or post-traumatic growth) [116, 117]. Although we found that mediating roles are evident for sense of coherence [78], this finding was derived from cross-sectional design. Nevertheless, Fig 2 provides a framework for testing mediating roles of personality on HRQOL, and adjudication of meaningful mediating effects can be further supported by datasets containing changes in health, personality, and HRQOL.

When studying the association of personality and HRQOL outcomes, caution should be taken with respect to several methodological and practical issues. First, a perennial problem in personality research is that the personality measures labeled the same name may capture different dimensions and the measures labeled different names may capture the same dimension. Several of the traits that we deem single can be explained by the Five-Factor Model. For example, type D is little more than high neuroticism and low extraversion; and aggressiveness is high neuroticism, low agreeableness, and low conscientiousness. To distinguish between single traits and personality dimensions, it is important to conduct additional analyses, for example calculating Pearson’s correlation to list a micro-trait (e.g., optimism) under a macro-dimension (e.g., neuroticism) if the magnitude exceeds a threshold (e.g., coefficient ≥0.4). Unfortunately, the majority of the papers we reviewed did not provide the intercorrelations that are needed to separate putative single traits from personality dimensions. Therefore, the approach we have taken may potentially inflate or deflate the magnitude of our findings.

Second, the relationships between personality characteristics and HRQOL can be partly attributed to the overlap in operationalization of the constructs and scale items. For example, extraversion is characterized by positive affect, and neuroticism is characterized by negative affect. This makes the relationship between neuroticism and reduced HRQOL somewhat tautological [103]. This argument may also help to explain why personality characteristics are more strongly associated with psychosocial aspects of HRQOL and share more variance in the construct than physical aspects of HRQOL. Kressin et al suggested the adjustment for symptoms of depression or anxiety as a strategy when investigating relationships between personality and HRQOL [77]. This approach helps partition out the common effect of disturbances in mood or other symptoms of affective disorder [60, 77].

Third, the extent to which dimensions of personality and HRQOL are congruent will influence the observed associations. Wasylkiw et al. suggested that the associations between personality and HRQOL may be more interpretable if specific aspects of personality dimensions (e.g. impulsiveness, angry hostility, self-consciousness) were matched with specific aspects of HRQOL [66]. Our systematic review sheds light on which personality traits are related in important ways to specific aspects of HRQOL.

Fourth, the practical application of existing standard personality measures is limited by their length. Most personality measures included in our review were lengthy; for example, 57 items in the EPI [79], and 88 items in the EPQ [81]. These measures are useful for psychological research, but may be cumbersome for clinical research and application. One possible solution is to select one or several important dimensions to assess. Another solution is to use short form measures that retain complex dimensions of personality characteristics, with acceptable psychometric properties. Some sample measures include the Midlife Development Inventory (MIDI) Personality Scale (25 items) [64], the Health-relevant Personality Inventory (HP5i) (20 items) [118], and the Ten-Item Personality Inventory (TIPI) [119].

Fifth, personality characteristics might contribute to the phenomena of response shift in HRQOL. Response shift is initially defined as the change of people’s internal standard or expectation in describing HRQOL concepts or interpreting HRQOL items after the occurrence of interventions or major events such as cancer [120, 121]. Rapkin and Schwartz introduced the concept of appraisal into the response shift framework and divided response shift into direct and moderated components [122, 123]. Direct response shift means the changes in appraisal affect HRQOL rating directly, as a result of personality characteristics relating to set-point maintenance after a change of health state. Moderated response shift means the changes in appraisal affect HRQOL ratings by attenuating the impact of health state changes. However, the effects of personality characteristics on response shift in HRQOL have not been fully investigated. One study found that cancer patients with pessimistic traits may report less improvement in HRQOL than patients with optimistic traits over time despite similar change in underlying health status [124]. Another study using advanced psychometric methods found that cancer patients were susceptible to response shift in general health assessment related to optimism [125].

Finally, this study is restricted to publications prior to December 31^st^, 2009. We have surveyed publications since 2009 and found that the current review covered a comprehensive list of personality and HRQOL measures and that the observed trends would not altered by adding these additional publication. Therefore, we believe that updating the literature would not change our conclusions significantly. Additionally, findings derived from this review study relied on a crude pooled estimate instead of a meta-analysis because only few studies were available for synthesizing the association with each of the specific personality traits and specific HRQOL domains in each meta-analysis (Table 2). However, the snapshot reported in this review provides a foundation for implementing future meta-analyses of the association of a specific personality trait with a specific HRQOL domain when a sufficient number of studies are available.

Understanding individual patients’ personality characteristics may help clinicians manage patients’ behaviors toward successful treatment adherence and health outcomes. Although a person’s personality characteristics are difficult to alter [126–129], it is possible to ameliorate or buffer the effect of personality on HRQOL by providing individualized treatment, counseling, or enhancing patients’ coping skill based on their unique personality characteristics. There are increasing interests in directing interventions at the processes through which personality is expressed in behavior and HRQOL. For example, strategies for illness appraisal and coping can be targeted, based on personality characteristics, with the goal of improving HRQOL [130]. Understanding the relationships of personality characteristics to HRQOL may be able to improve patient self-management. It is also possible self-knowledge by patients of their own personality characteristics and the predictable relationship of these characteristics to their well-being may influence their proclivity to adopt specific, targeted interventions.

An important next step for research is to apply pathway approaches [130] to explain the mechanism by which personality characteristics lead to important outcomes (e.g., behaviors, longevity, HRQOL, well-being) over the life course [131]. If personality characteristics confound the relationships between variables of interest (such as treatment regimens) and HRQOL outcomes, personality variables should be collected and accounted for in study design or statistical models. All of this suggests that it may be useful for clinicians and researchers to collect and identify individuals’ personality characteristics to better understand and interpret subsequent HRQOL.

The clinical and research significance of the relationships between personality and HRQOL suggest further implications for policy and practice. We believe that personality should be measured more routinely in clinical practice, as well as for clinical and health care research. There is the opportunity to introduce personality measures into the electronic health record as a form of patient-generated data, using patient portals and other electronic collection methods. Doing so would make information on personality characteristics available for multiple applications, and to generate new evidence for the usefulness of these data [132].

## Conclusions

In conclusion, personality characteristics at the levels of domain or individual trait, respectively, are associated with HRQOL at overall, physical, psychological, social, and other domains, respectively. However, the magnitudes for the corresponding associations are different. Specifically, we found that personality is more often related to psychosocial aspects of HRQOL than to physical aspects, and personality traits such as neuroticism and negative affectivity are strongly associated with mental aspects of HRQOL. Personality characteristics had indirect, mediating, and moderating effects on different aspects of HRQOL. Interpreting these relationships is complicated by overlap in how the concepts of personality and HRQOL are operationalized. Future research is needed to distinguish among the various constructs and measures of personality and HRQOL. The thoughtful and systematic collection of personality data could be useful for both research and clinical practice.

## Supporting information

S1 Checklist PRISMA checklist. (DOCX)

S1 Appendix Definition of personality characteristics. (DOCX)
