# Supplementary material for: Does personality affect health-related quality of life? A systematic review
Source: PLoS One. 2017 Mar 29;12(3):e0173806. doi: 10.1371/journal.pone.0173806 (PMC5371329; doi:10.1371/journal.pone.0173806)
Supplement: S1 Appendix — (DOCX) [file pone.0173806.s002.docx]

S1 Appendix: Definition of personality characteristics

| Personality dimension | Definition | Trait | Measure | Reference |
| --- | --- | --- | --- | --- |
| Agreeableness | The quality of interpersonal orientation along a continuum from compassion to antagonism in thoughts, feelings, and actions | - HiPIC: altruism, dominance, egocentrism, compliance, and irritability - MIDI: helpful, warm, caring, softhearted, sympathetic - NEO-PI/NEO-PI-R/NEO-FFI: trust, straightforwardness, altruism, compliance, modesty, tender-mindedness | Hierarchical Personality Inventory for Children (HiPIC), Midlife Development Inventory Personality Scales (MIDI), NEO Personality Inventory (NEO-PI), NEO Five Factor Inventory (NEO-FFI) | [1-3] |
| Conscientiousness | The degree of organization, persistence, and motivation in goal-directed behavior | - HiPIC: concentration, perseverance, orderliness, and achievement motivation - MIDI: organized, responsible, hardworking, (not) careless - NEO-PI/NEO-PI-R/NEO-FFI: competence, order, dutifulness, achievement striving, self-discipline, deliberation | Hierarchical Personality Inventory for Children (HiPIC), Midlife Development Inventory Personality Scales (MIDI), NEO Personality Inventory (NEO-PI), NEO Five Factor Inventory (NEO-FFI) | [1-3] |
| Extraversion | The quantity and intensity of interpersonal interaction, activity level, need for stimulation, and capacity for joy | - EPI/EPQ: sociability, liveliness, activeness, assertiveness, sensation seeking, dominance, being adventuresome - HiPIC: energy, expressiveness, optimism, and shyness - MIDI: outgoing, friendly, lively, active, talkative - NEO-PI/NEO-PI-R/NEO-FFI: warmth, gregariousness, assertiveness, activity, excitement seeking, positive emotions | Eysenck Personality Inventory (EPI), Eysenck Personality Questionnaire (EPQ), Hierarchical Personality Inventory for Children (HiPIC), Midlife Development Inventory Personality Scales (MIDI), NEO Personality Inventory (NEO-PI), NEO Five Factor Inventory (NEO-FFI) | [1-6] |
| Neuroticism | A tendency to psychological distress, unrealistic ideas, excessive cravings or urges, and maladaptive coping responses | - EPI/EPQ: anxiety, hostility, depression, self-esteem , Impulsiveness, vulnerability - HiPIC: anxiety, self-confidence - MIDI: moody, worrying, nervous, (not) calm - NEO-PI/NEO-PI-R/NEO-FFI: anxiety, depression, guilt feeling, low self-consciousness, tension, shyness, being emotional - DPI: neuroticism | Eysenck Personality Inventory (EPI), Eysenck Personality Questionnaire (EPQ), Hierarchical Personality Inventory for Children (HiPIC), Midlife Development Inventory Personality Scales (MIDI), NEO Personality Inventory (NEO-PI), NEO Five Factor Inventory (NEO-FFI), Dutch Personality Inventory (DPI) | [1-7] |
| Openness to experience | The proactive seeking and appreciation of experience for its own sake, toleration for and exploration of the unfamiliar | - HiPIC: creativity, intellect, and curiosity - MIDI: creative, imaginative, intelligent, curious, sophisticated, adventurous - NEO-PI/NEO-PI-R/NEO-FFI: fantasy, aesthetics, feelings, actions, ideas, values | Hierarchical Personality Inventory for Children (HiPIC), Midlife Development Inventory Personality Scales (MIDI), NEO Personality Inventory (NEO-PI), NEO Five Factor Inventory (NEO-FFI) | [1-3] |
| Psychoticism | A tendency or a predisposition associated with non-conformity, hostility, and impulsivity | Aggressiveness, coldness, egocentricity, being impersonal, impulsiveness, antisocial, creative, tough-mindedness | Eysenck Personality Inventory (EPI), Eysenck Personality Questionnaire (EPQ) | [1, 3-6] |
| Single personality trait | Definition | | Measure | Reference |
| Agency | A focus on the self, including autonomy, self-advancement, and separation from others | | Personal Attributes Questionnaire (PAQ) | [8] |
| Aggression | An intentional attempt to harm another person | | Buss-Perry Aggression Questionnaire (BPA) | [9] |
| Alexithymia | Difficulties in experiencing, differentiating, and verbalizing feelings | | Toronto Alexithymia Scale (TAS) | [10] |
| Communion | A focus on other people and relationships | | Personal Attributes Questionnaire (PAQ),  Observer Alexithymia Scale (OAS) | [8, 11] |
| Lie | A tendency to try to look better than one actually is | | Eysenck Personality Inventory (EPI), Eysenck Personality Questionnaire (EPQ) | [4, 6] |
| Dispositional optimism | A global expectation that good things will be plentiful in the future and bad things will be scarce | | Life Orientation Test (LOT), The Assessment of Beliefs in Self-Efficacy and Optimism (SWOP) | [12, 13] |
| Hopefulness | A general tendency to construct and respond to the perceived future positively | | Hunter Opinions and Personal Expectations Scale (HOPES) | [14] |
| Mastery | The feeling as the extent to which a person perceives himself or herself to be in control of events and ongoing situations | | The Pearlin Mastery Scale | [15] |
| Negative affectivity | A general disposition to subjective distress, including anger, scorn, guilt, and depression | | The short form of Eysenck Personality Inventory Emotional Stability Scale (EPI-Q) | [16] |
| Sense of coherence | A feeling of confidence that one’s internal and external environment are predictable and that there is a high probability that things will work out as well as can reasonably be expected | | The Sense of Coherence (SOC) Scale | [17] |
| Self-efficacy | A person’s belief or sense of confidence in one’s own ability to perform a particular task or behavior successfully | | Self-Efficacy Scale (SES) | [18, 19] |
| Self-esteem | A favorable or unfavorable attitude toward the self | | Rosenberg Self-Esteem Scale (RSE), |  |
| Trait anxiety | A stable tendency to respond with anxiety in the anticipation of threatening situations | | The State-Trait Anxiety Inventory (STAI) | [20, 21] |
| Type-D | Chronic emotional distress (i.e., a tendency to experience negative emotions across time/situation and inhibit emotions and behaviors in social interaction) | | Type D Scale 14 (DS14), Type D Scale 16 (DS16), Type D Scale 24 (DS 24) | [22-24] |
| Unmitigated communion | An extreme focus on others and a lack of focus on the self | | Helgeson’s Revised Unmitigated Communion Scale, Personal Attributes Questionnaire (PAQ) | [25] |

**References:**

1. Costa, P. T., & McRae, R. R. (1985). *The NEO Personality Inventory Manual*. Odessa, FL: Psychological Assessment Resources.

2. Costa, P. T., & McCrae, R. R. (1992). *Revised NEO Personality Inventory (NEO PI-R) and Neo Five-Factor Inventory (NEO-FFI)*. Odessa, FL: Psychological Assessment Resources.

3. De Fruyt, F., Bartels, M., Van Leeuwen, K. G., De Clercq, B., Decuyper, M., & Mervielde, I. (2006). Five types of personality continuity in childhood and adolescence. *Journal of Personality and Social Psychology*, *91*(3), 538–552.

4. Eysenck, H. J., & Eysenck, S. B. G. (1975). *Manual of the Eysenck Personality Questionnaire (Junior and Adult)*. Kent, UK: Hodder and Stoughton.

5. Eaves, L. J., Eysenck, H. J., Martin, N. G., Jardine, R., Heath, A. C., Feingold, L., et al. (1989). *Genes, culture and personality: An empirical approach*. Cambridge, UK: Cambridge University Press.

6. Eysenck, H. J. (1991). Dimensions of personality: 16, 5 or 3?—Criteria for a taxonomic paradigm. *Personality and Individual Differences*, *12*(8), 773–790.

7. Luteyn, F., Starren, J., & Van Dijk, J. (1985). *Manual for the Dutch Personality Questionnaire*. Amsterdam: Swets & Zeitlinger.

8. Bakan, D. (1966). *The duality of human existence: An essay on psychology and religion*. Chicago, IL: Rand McNally.

9. Buss, A. H., & Perry, M. (1992). The aggression questionnaire. *Journal of Personality and Social Psychology*, *63*(3), 452–459.

10. Taylor, G. J., Bagby, R. M., Ryan, D. P., Parker, J. D., Doody, K. F., & Keefe, P. (1988). Criterion validity of the Toronto Alexithymia Scale. *Psychosomatic Medicine*, *50*(5), 500–509.

11. Haviland, M. G., Warren, W. L., & Riggs, M. L. (2000). An observer scale to measure alexithymia. *Psychosomatics*, *41*(5), 385–392.

12. Scheier, M. F., & Carver, C. S. (1985). Optimism, coping, and health: assessment and implications of generalized outcome expectancies. *Health Psychology*, *4*(3), 219–247.

13. Scholler, G., Fliege, H., & Klapp, B. F. (1999). Fragebogen zu Selbstwirksamkeit, Optimismus und Pessimismus: Restrukturierung, Itemselektion und Validierung eines Instrumentes an Untersuchungen klinischer Stichproben [Questionnaire for self-efficacy, optimism and pessimism: Reconstruction, selection of items and validation of an instrument by means of examinations of clinical samples]. *Psychotherapie, Psychosomatik, Medizinische Psychologie*, *49*(8), 275–283.

14. Nunn, K. P., Lewin, T. J., Walton, J. M., & Carr, V. J. (1996). The construction and characteristics of an instrument to measure personal hopefulness. *Psychological Medicine*, *26*(03), 531–545.

15. Pearlin, L. I., & Schooler, C. (1978). The Structure of Coping. *Journal of Health and Social Behavior*, *19*(1), 2. doi:10.2307/2136319

16. Floderus, B. (1974). *Psycho-social factors in relation to coronary heart disease and associated risk factors*. Stockholm: Department of Environmental Hygiene of the Karolinska Institute.

17. Antonovsky, A. (1987). *Unraveling the mystery of health: How people manage stress and stay well*. San Francisco, CA: Jossey-Bass Publishers.

18. Kobayashi, M., Ohno, T., Noguchi, W., Matsuda, A., Matsushima, E., Kato, S., & Tsujii, H. (2009). Psychological distress and quality of life in cervical cancer survivors after radiotherapy: do treatment modalities, disease stage, and self-esteem influence outcomes? *International Journal of Gynecological Cancer*, *19*(7), 1264–1268. doi:10.1111/IGC.0b013e3181a3e124

19. Middleton, J., Tran, Y., & Craig, A. (2007). Relationship Between Quality of Life and Self-Efficacy in Persons With Spinal Cord Injuries. *Archives of Physical Medicine and Rehabilitation*, *88*(12), 1643–1648. doi:10.1016/j.apmr.2007.09.001

20. Rosenberg, M. *Society and the Adolescent Self Image.* Princeton, NJ; 1965. Princeton University Press.

21. Spielberger, C. D., Gorsuch, R. L., Lushene, R. E., Vagg, P. R., & Jacobs, G. A. (1983). *Manual for the State-Trait Anxiety Inventory*. Palo Alto, CA: Consulting Psychologists Press.

22. Denollet, J., Vaes, J., & Brutsaert, D. L. (2000). Inadequate response to treatment in coronary heart disease: adverse effects of type D personality and younger age on 5-year prognosis and quality of life. *Circulation*, *102*(6), 630–635.

23. Denollet, J. (1998). Personality and coronary heart disease: the type-D scale-16 (DS16). *Annals of Behavioral Medicine*, *20*(3), 209–215.

24. Denollet, J. (2005). DS14: standard assessment of negative affectivity, social inhibition, and Type D personality. *Psychosomatic Medicine*, *67*(1), 89–97.

25. Helgeson, V. S., & Fritz, H. L. (2000). The implications of unmitigated agency and unmitigated communion for domains of problem behavior. *Journal of Personality*, *68*(6), 1031–1057.
